# Postoperative Rehabilitation Program for Increasing Muscle Mass in Patients With Hip Fracture: A Retrospective Study

**DOI:** 10.7759/cureus.63053

**Published:** 2024-06-24

**Authors:** Marina Sajiki-Ito, Shinji Tsukamoto, Daisuke Bai, Mitsunori Tokuda, Katsuya Tamai, Naoki Takeguchi, Masayuki Sada, Yasuhito Tanaka, Akira Kido

**Affiliations:** 1 Department of Rehabilitation Medicine, Nara Medical University, Nara, JPN; 2 Department of Orthopaedic Surgery, Nara Medical University, Nara, JPN; 3 Graduate School of Medicine, Health and Science, Kio University, Nara, JPN; 4 Rehabilitation, Yokohama Sports Medical Center, Kanagawa, JPN; 5 Department of Rehabilitation, Heisei Memorial Hospital, Nara, JPN; 6 Department of Orthopaedic Surgery, Heisei Memorial Hospital, Nara, JPN

**Keywords:** neuromuscular electrical stimulation, bioelectrical impedance analysis, rehabilitation program, hip fracture, muscle mass

## Abstract

Background: Hip fractures are most likely to occur in older people, and after hip surgery muscle mass and the ability to perform activities of daily living often decline. In this study, we conducted inpatient rehabilitation after surgery for hip fracture and measured changes in muscle mass and physical performance.

Methods: We retrospectively analyzed patients aged 65 years or older who underwent surgery (prosthetic replacement or internal fixation) and inpatient rehabilitation for hip fracture at our hospital between August and December 2020. The training included a joint range of motion exercise, muscle-strengthening exercise, gait training, early mobilization training, and neuromuscular electrical stimulation. We measured the following factors after one and six weeks postoperatively: muscle mass, body weight, fat mass, grip strength, bilateral knee extension strength, ability to walk, and ability to perform activities of daily living.

Results: Seventeen patients were included. Median age was 84 years (interquartile range, 72-90). Lower limbs skeletal muscle mass increased (median 4.8 kg to 4.9 kg, p = 0.045), while upper limbs skeletal muscle mass and body weight decreased (median 1.2 kg to 1.1 kg, p = 0.0027), (median 46.8 kg to 45.5 kg, p = 0.0039), respectively. Total skeletal muscle mass and fat mass remained unchanged. Grip strength was maintained, and knee extension muscle strength on the healthy and affected sides increased (healthy side median 10.7 kgf to 13.7 kgf, p = 0.019; affected side median 5.5 kgf to 9.5 kgf, p < 0.001). All patients exhibited improved ability to perform activities of daily living; however, 52.9% of patients regained their pre-injury walking ability.

Conclusions: Our rehabilitation program increased lower limb skeletal muscle mass in patients with hip fractures.

## Introduction

The number of patients with hip fractures continues to increase with the growth of the elderly population worldwide. Hip fractures are increasing exponentially among people over 70 years of age, and the number of patients with hip fractures in Japan was approximately 190,000 per year in 2012; however, by 2040 it is estimated that the number will reach approximately 300,000 people [1].

Patients with hip fractures are often elderly and include individuals with cognitive decline and systemic complications at the time of injury; many of these patients exhibit difficulty with activities of daily living (ADL) [2]. Additionally, it has been reported that approximately half of hip fracture patients have impairment in ADL postoperatively [3]. Thus, the decline in the ability to perform ADL is a problem after surgery for a hip fracture. Postoperative rehabilitation is considered useful, not only for improving the ability to perform daily activities but also to prevent postoperative complications [3,4]. Additionally, previous studies have reported that rehabilitation also improved muscle strength and shortened the length of hospital stay [4]. One of the key goals of rehabilitation is to enable the patient to stand or walk. Specifically, it is recommended that lower limb muscle strengthening and range-of-motion training begin the day after surgery and that gait training progresses from parallel bars to walkers to crutches, to walking with a T-cane [4,5].

It is also considered important to focus on increasing muscle mass as an indicator of the effectiveness of rehabilitation because low muscle mass makes patients more prone to falls, which can lead to the need for nursing care [6]. Furthermore, it has been reported that as well as decreasing the ability to perform ADL and the rate of home discharge, low muscle mass also increases mortality rates [7]. Some previous studies reported a decrease in limb skeletal muscle mass and the ability to perform ADL during the first year of postoperative follow-up [8].

Therefore, postoperative rehabilitation to increase muscle mass is considered to be necessary for patients with hip fractures. However, when examining previous reports on measuring skeletal muscle mass in patients undergoing rehabilitation after surgery for hip fracture, some reported that skeletal muscle mass increased, while others showed it decreased. Of these, two studies reported an increase in skeletal muscle mass. However, since both studies were conducted several weeks after surgery, detailed rehabilitation protocols immediately after surgery and their effects were unknown [5,6,8-13].

In this study, we measured muscle mass and ADL over time in patients who underwent rehabilitation from the day after surgery for a hip fracture and examined the effects of our rehabilitation program.

## Materials and methods

We retrospectively analyzed patients aged 65 years or older who underwent surgery (prosthetic replacement or internal fixation) and inpatient rehabilitation for hip fracture at our hospital between August and December 2020. Patients with fracture of the femoral head, greater or lesser trochanter only, inferior trochanter, or pathological fracture, those who were discharged or transferred within six weeks after surgery, and those who could not continue rehabilitation because of poor general condition were excluded [14]. This study was approved by the Heisei Memorial Hospital Ethics Committee (protocol code 2019-002). The following baseline information was obtained from patients’ medical records: age, gender, height, weight, body mass index, comorbidities, fracture type, surgical procedure, surgical waiting time, and hospital stay. Comorbidities were also assessed using the Charlson comorbidity score [15].

Rehabilitation program

In the Japanese medical system, there are two types of wards: “acute care wards,” which provide intensive medical care aimed at early stabilization of conditions, and “convalescent wards,” which provide intensive rehabilitation to patients who have passed the acute stage of illness and help them improve their ability to perform ADL and return home. Although many hospitals have only one type of ward, because our hospital has both an acute care ward and a convalescent ward, we were able to provide consistent treatment and rehabilitation from admission to discharge to home or nursing home. Patients with fractures were admitted to the acute care ward first, and when their general condition was stabilized after surgery, they were transferred to the convalescent wards. The rehabilitation program set by the government differed between the acute ward and the convalescent ward. We were able to provide training at the acute ward for up to two hours per day and up to three hours per day at the convalescent ward, seven days a week in both wards, following the Japanese medical system.

**Figure 1:**
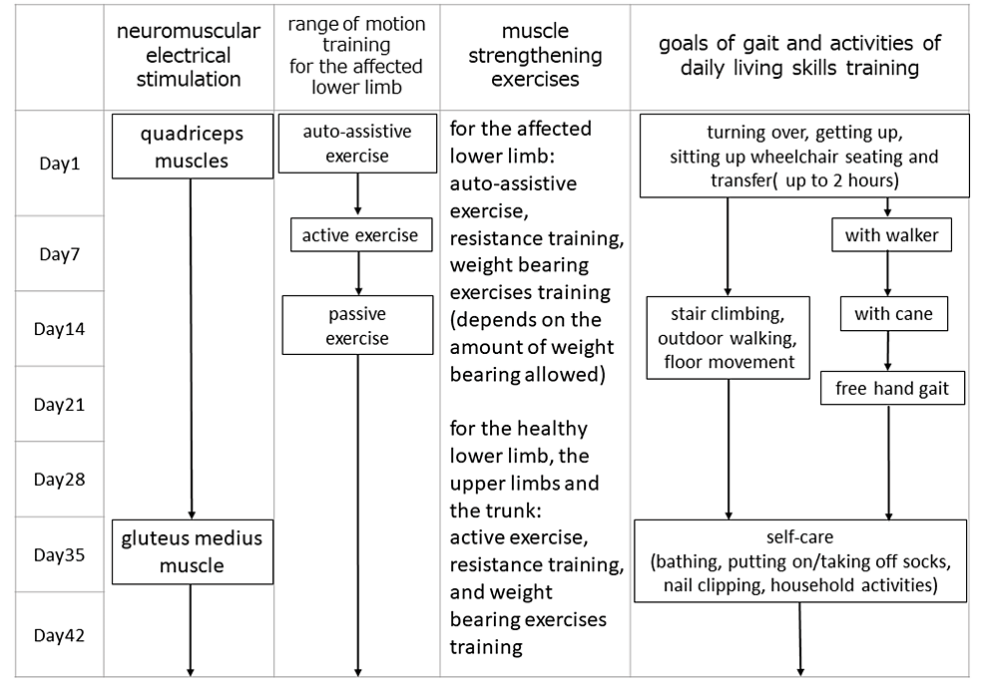
Our rehabilitation program The program is characterized by aggressive early mobilization training and neuromuscular electrical stimulation.

The training was based on our rehabilitation program for hip fractures, with modifications according to the patient’s condition, especially weight-bearing restrictions. Our rehabilitation program is shown in Figure 1. Our program was characterized by two points: first, we actively conducted early mobilization training, and second, we used neuromuscular electrical stimulation [16,17]. Regarding early mobilization training, wheelchair transfer training was conducted in the patient’s room and training room from the first day or two after surgery. Patients were then trained to walk with a walker one week after surgery and to walk with a cane two weeks after surgery. In addition, although it depended on the patient’s condition, to ensure that patients had time to get out of bed even outside of training hours, they ate in a wheelchair-sitting position as much as possible in their rooms and performed gait training with nurses.

**Figure 2:**
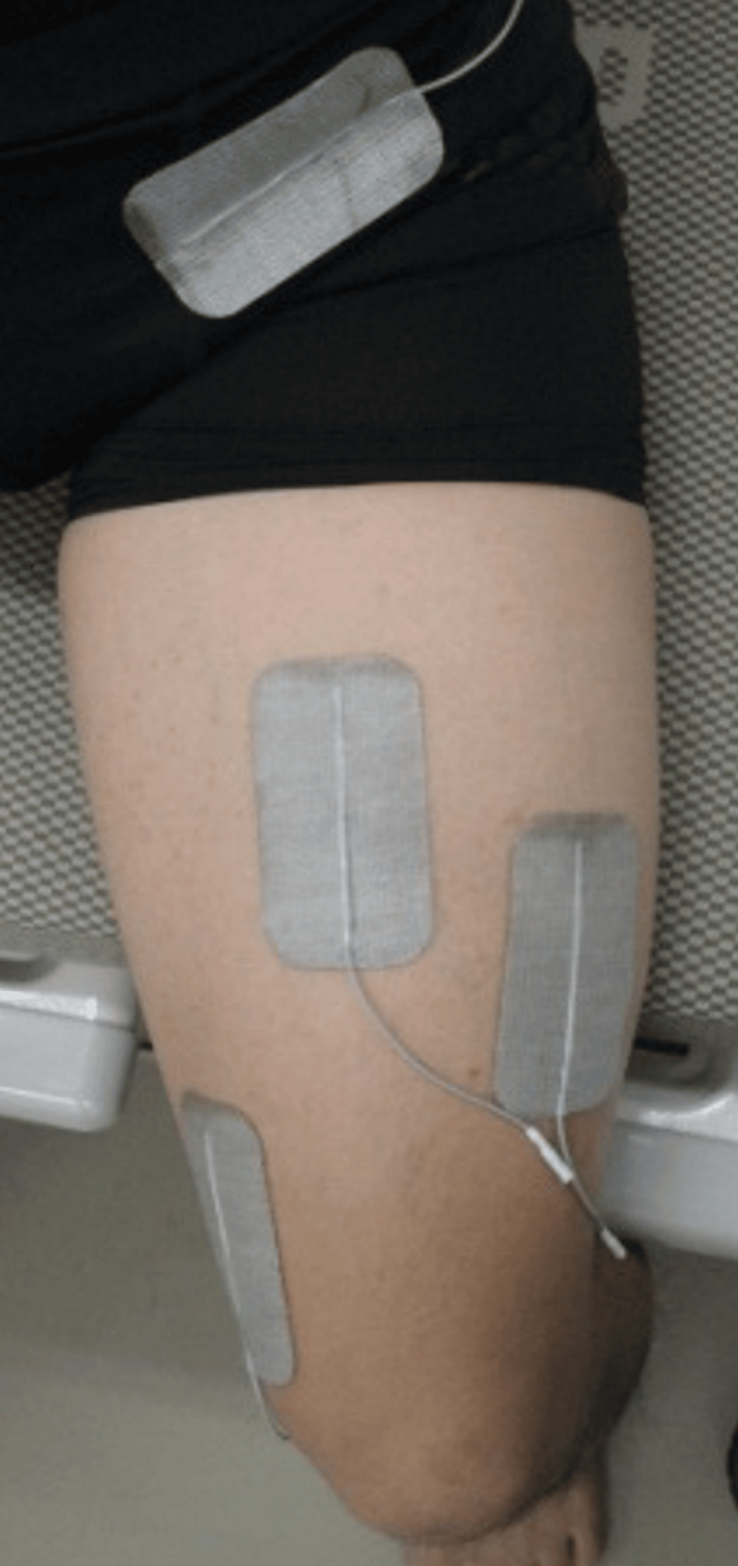
The location of the quadriceps stimulating electrode in neuromuscular electrical stimulation The electrodes were placed just above the femoral nerve and at the motor points of the rectus femoris, vastus medialis, and vastus lateralis muscles.

**Figure 3:**
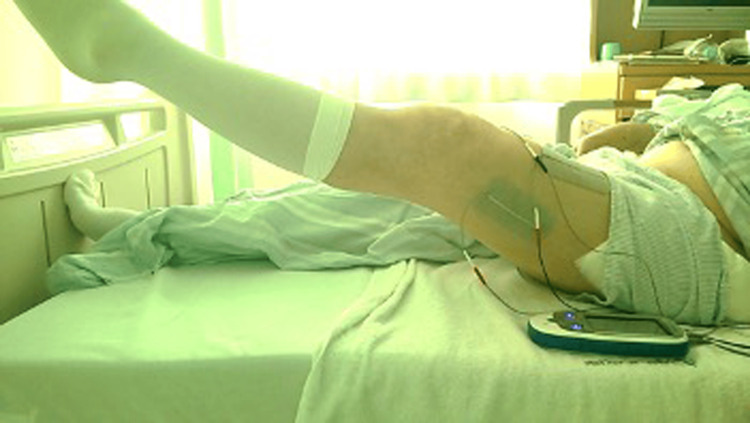
Knee extension training combined with neuromuscular electrical stimulation

Neuromuscular electrical stimulation was performed on the affected lower limb in all patients from the day after surgery using ESPURGE (ITO physiotherapy & rehabilitation, Japan). Specifically, from the day after surgery to the fourth week, the patient underwent a combination of electrical stimulation targeting the quadriceps muscles (rectus femoris, vastus medialis, and vastus lateralis) and voluntary knee extension exercises. Depending on the patient’s condition, electrical stimulation was performed for a maximum of 20 minutes, and a total of 100 knee extension exercises were performed (Figures 2, 3). For five to eight weeks postoperatively, electrical stimulation of the gluteus medius muscle was combined with voluntary hip abduction and adduction exercises in the supine position, one-leg standing, and gait training for a maximum of 20 minutes, depending on the patient’s condition. In addition, muscle strengthening exercises for the limbs and trunk (active or passive exercise, resistance training, and weight-bearing exercises training), range of motion training for the affected lower limb, and daily living exercises were also provided from the day after surgery, as needed.

Outcomes

**Figure 4:**
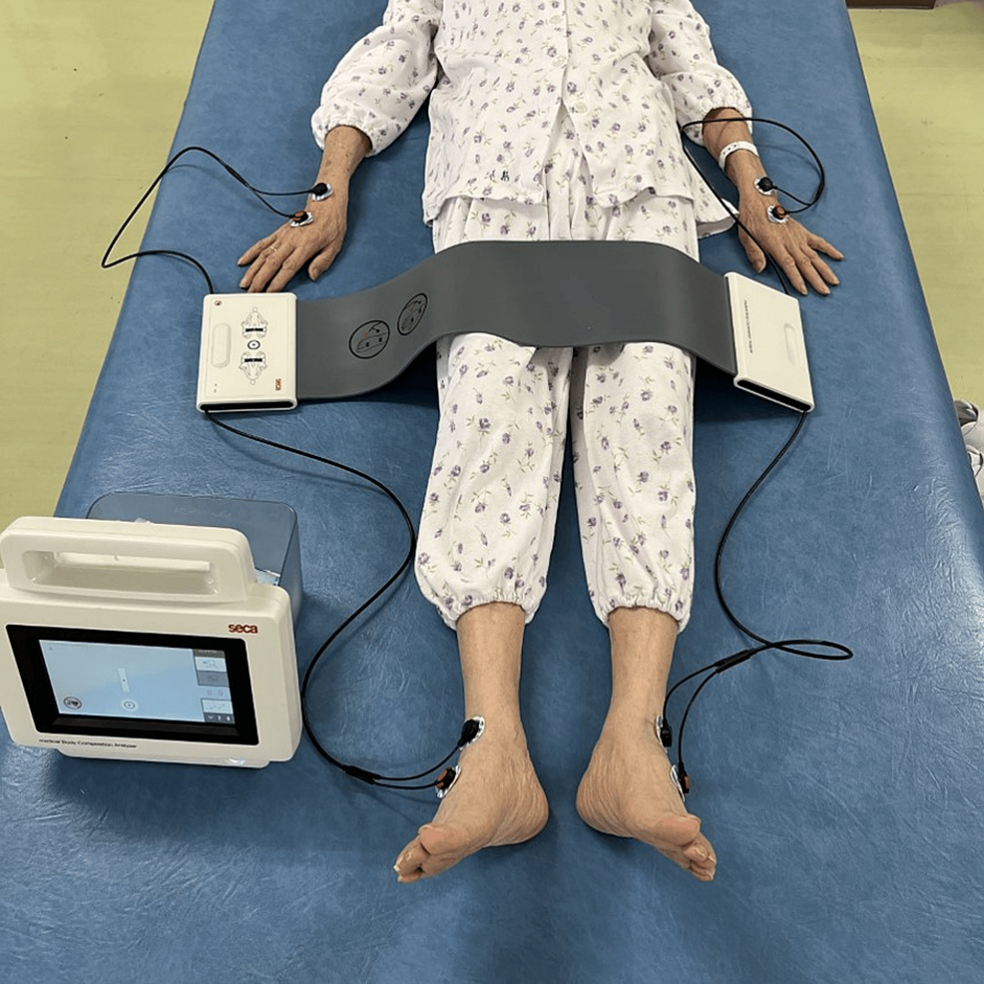
SECA mBCA515 measurement in the supine position. A resting period of 20 minutes was allowed before the measurements were conducted, in accord with recommended use. All measurements were performed in the supine position. Two electrodes were placed on each limb (hands and feet) at a distance of 5 cm. SECA mBCA515 device (Seca GmbH & Co. KG, Hamburg, Germany).

We investigated body composition (skeletal muscle mass, body weight and fat mass), muscle strength (grip strength and knee extension strength), walking ability, and ability to perform ADL as outcomes. A SECA mBCA515 (Seca GmbH & Co. KG, Hamburg, Germany) device was used to measure body composition (Figure 4). This is an eight-point contact electrode method, multi-frequency bioelectrical impedance analysis instrument that delivers electric current from the distal extremities [18]. A resting period of 20 minutes was set before measurements according to the recommended usage. All measurements were performed in the supine position. Two electrodes were placed on each limb at a distance of 5 cm. The electrode sites were cleaned before the electrodes were attached. Measurements included SMM of total body, trunk, and bilateral upper and lower limbs, body weight, and fat mass. The grip strength of the dominant hand and bilateral knee extension muscle strength were measured as indices of muscle strength. Grip strength was measured using a hand grip dynamometer (Takei Scientific Instruments Co., Ltd, Nigata, Japan). Patients held the hand grip dynamometer with their dominant hand at maximum force for at least 5 seconds in a seated position with the upper limb drooped, and maximum grip strength was recorded as the highest value of two trials [19]. Knee extension muscle strength was measured using a portable dynamometer, μ-tas F-1 (Animaco., Ltd, Tokyo, Japan) with the patient in a sitting position with the knee flexed to 90°. The patient was instructed to gradually increase the intensity of knee extension to the dynamometer for approximately 2 seconds while avoiding explosive extension and to maintain maximum muscle output for approximately 3 seconds. Measurements were taken twice from the bilateral lower limbs, and the maximum value was recorded for each [20]. Walking ability was measured using the Functional Ambulation Categories instrument [21,22]. The minimum score was 0 and the maximum was 5. The pre-injury status was obtained from the patient or family, and at six weeks postoperatively, the patient’s physical therapist conducted an evaluation. The ability to perform ADL was assessed using the Functional Independence Measure (FIM) [23] and motor FIM score, representing motor items, was recorded. Assessments were performed by the physical therapist in charge at admission, after one and six weeks postoperatively, and just before discharge.

Statistical analysis

The Shapiro-Wilk test was used to assess and examine normal distribution. Because all of the anthropometric measurements (muscle mass, body weight, and fat mass) and muscle strength (grip strength and knee extension strength) were non-parametric, Wilcoxon’s signed rank sum test was used. Measures of non-parametric data were expressed as median and interquartile range (IQR). Statistical significance was set at p < 0.05. All of the analyses were performed using JMP 14 software (SAS Institute Inc., Cary, NC, USA).

## Results

Thirty-five patients met the recruitment criteria and 17 were observed until the final follow-up period (six weeks after surgery). Of the 18 patients who dropped out, two were unable to continue rehabilitation due to deterioration in their general condition. Two were discharged home before six weeks. Fourteen patients were transferred to another rehabilitation hospital. The median postoperative follow-up period for the 17 patients was 68 days (IQR, 53-98). Table 1 shows the basic data of the 17 patients on admission. Four patients were male, and 13 were female, with a median age of 84 years (IQR, 72-90). The median Charlson comorbidity score was 2 (IQR, 0.5-2.0). Eleven patients were diagnosed with neck fracture and six patients were diagnosed with trochanteric fracture. Eleven patients underwent prosthetic replacement, and six underwent internal fixation (Table 2). Details of fracture types and surgical procedures are shown in Table 2. The median waiting time for surgery was six days (IQR, 4.5-8.5), and the median length of stay in the acute care and recovery wards was 19 days (IQR, 15.5-28.5) and 55 days (IQR, 33.5-77), respectively (Table 1).

**Table 1:** Participants’ characteristics Results are expressed as median (interquartile range, IQR). n, %: Number of patients, percentage. BMI: Body mass index. FAC: Functional ambulation categories. FIM: Functional independence measure.

Age (years)	Median 84 (IQR 72–90)
Female, n (%)	13 (76.5%)
Height (m)	Median 1.5 (IQR 1.47–1.58)
Weight (kg)	Median 46.8 (IQR 43.6–53.3)
BMI (kg/m^2^)	Median 22.2 (IQR 19.4–23.2)
Charlson comorbidity index	Median 2 (IQR 0.5–2.0)
Type of fracture	
Neck fracture (n)	11
Trochanteric (n)	6
Surgical method	
Prosthetic replacement (n)	6
Internal fixation (n)	11
Time to surgery (days)	Median 6 (IQR 4.5–8.5)
Length of stay (days)	Median 68 (IQR 53–98)
Acute wards (days)	Median 19 (IQR 15.5–28.5)
Convalescent ward (days)	Median 55 (IQR 33.5–77.0)
FAC (n)	
5	7
4	4
3	3
2	2
1	0
0	1
Motor FIM score	Median 18 (IQR 16.0–27.5)

**Table 2:** Number of patients by the type of hip fracture and surgical method n: Number of patients.

Neck fracture	(n) 11
Garden classification	
I	0
II	4
III	0
IV	7
Surgical method	
Bipolar hip arthroplasty	6
Screws	5
Trochanteric fracture	6
Evans classification	
Type 1 group 1	1
Type 1 group 2	0
Type 1 group 3	4
Type 1 group 4	1
Type 2	0
Surgical method	
Short femoral nail	2
Long femoral nail	4

**Figure 5:**
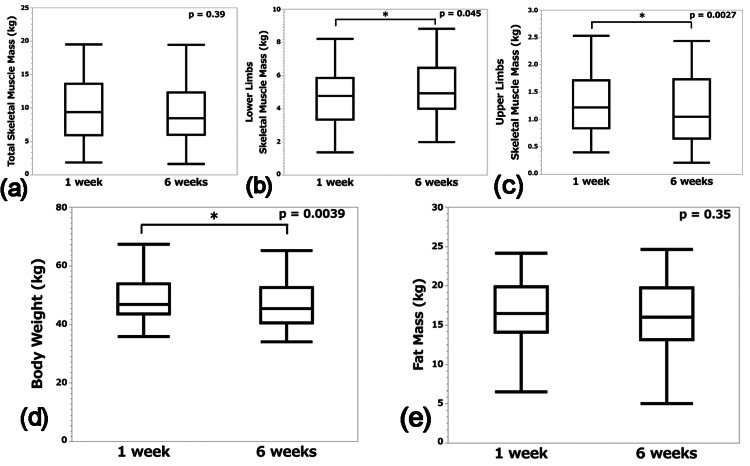
Body composition at 1 week and 6 weeks postoperatively. Box-and-whisker plots of total skeletal muscle mass (a), lower limbs skeletal muscle mass (b), upper limbs skeletal muscle mass (c), body weight (d), and fat mass (e) at one and six weeks postoperatively.

Skeletal muscle mass, body weight, and fat mass at one and six weeks postoperatively are shown in Figure 5. There was no change in total skeletal muscle mass (9.4 kg [IQR, 5.9-13.6] to 8.5 kg [IQR, 6.0-12.4], p = 0.39) (Figure 5a). However, lower limbs skeletal muscle mass increased (4.8 kg [IQR, 3.4-5.9] to 4.9 kg [IQR, 4.0-6.5], p = 0.045 (Figure 5b) and upper limbs skeletal muscle mass decreased (1.2 kg [IQR, 0.8-1.7] to 1.1 kg [IQR, 0.7-1.7], p = 0. 0027) (Figure 5c). Weight decreased (46.8 kg [IQR, 43.6-54.0] to 45.5 kg [IQR, 40.5-52.6], p = 0.0039) (Figure 5d) and there was no change in fat mass (16.5 kg [IQR, 14.1-19.9] to 16.0 kg [IQR, 13.2-19.7], p = 0.35) (Figure 5e).

**Figure 6:**
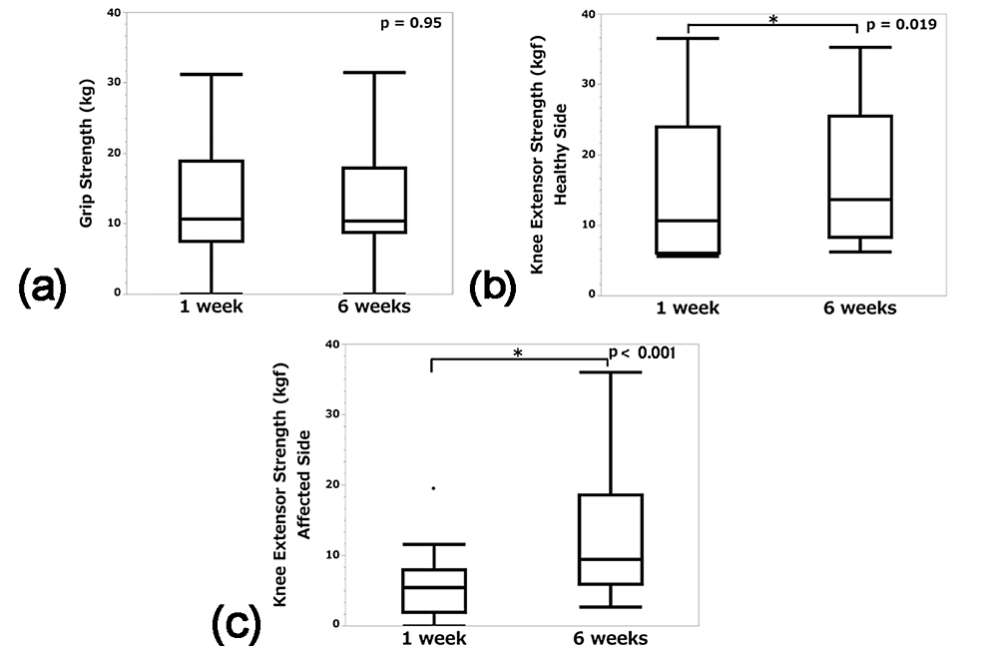
Muscle strength at one week and six weeks postoperatively Box-and-whisker plots of grip strength (kg) of the dominant hand (a) and knee extensor strength (kgf) of the healthy (b)/affected (c) side at one and six weeks postoperatively are shown.

The grip strength of the dominant hand and bilateral knee extension muscle strength at one and six weeks postoperatively are shown in Figure 6. There was no change in grip strength of the dominant hand (10.7 kg [IQR, 7.6-18.9] to 10.4 kg [IQR, 8.8-18.0], p = 0.95) (Figure 6a), while knee extensor strength increased on both the healthy and affected sides (healthy side 10.7 kgf [IQR, 6.1-24.0] to 13.7 kgf [IQR, 8.4-25.5], p = 0.019, Figure 6b), affected side 5.5 kgf [IQR, 2.0-8.0] to 9.5 kgf [IQR, 6.0-19.0], p < 0.001, Figure 6c).

**Figure 7:**
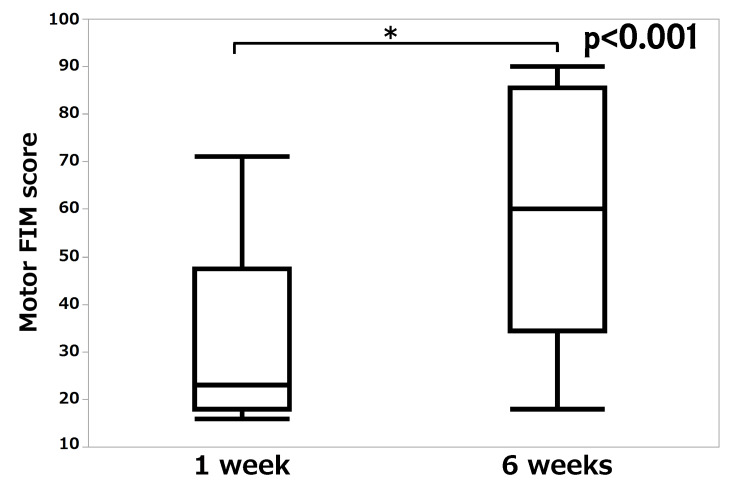
Motor FIM score at one week and six weeks postoperatively Motor Functional Independence Measure (FIM) scores at one week and six weeks postoperatively are shown in box-and-whisker plots. The horizontal line in the box represents the median value, and the lines above and below the box represent the minimum and maximum values.

Finally, Table 3 shows the change in walking ability using Functional Ambulation Categories. Seven patients were ambulatory independent (Functional Ambulation Categories score of 5) before admission, but only four patients were ambulatory independent at six weeks. At six weeks postoperatively, 52.9% (nine of 17 patients) exhibited recovery in walking ability that was equal to or better than that before admission. Motor FIM score had a median of 18 (IQR, 16-27.5) at admission, 23 (IQR, 18-47.5) at one week postoperatively, 60 (IQR, 34.5-85.5) at six weeks postoperatively, and 75 (IQR, 40.8-88) at discharge, with all patients exhibiting an increase over time from admission to discharge. Specifically, a comparison of scores at one and six weeks showed improvement (p < 0.001, Figure 7).

**Table 3:** Walking ability assessed with FACs Number of patients = 17. FAC: Functional ambulation categories, n: Number of patients.

FAC	Before admission (n)	6 weeks after surgery (n)
5	7	4
4	4	5
3	3	2
2	2	1
1	0	2
0	1	3

## Discussion

Eight previous studies reported changes in skeletal muscle mass in patients undergoing rehabilitation after hip fracture (Table 4) [5,6,8-13]. Of these studies, five reported no change, one study reported a decrease in muscle mass, and two studies reported an increase in muscle mass. The causes of the disparity in results among the eight studies to date are thought to be (1) differences in the measurement methods for body composition, (2) differences in the timing and duration of rehabilitation intervention, and differences in protocols, and (3) the presence or absence of concomitant use of nutritional therapy. Of the studies that found increased skeletal muscle mass, the current study is the first to report the effects of a detailed rehabilitation program immediately after surgery. In this study, we demonstrated an increase in lower limb skeletal muscle mass after six weeks of our rehabilitation program starting the day after surgery, which included early mobilization training and neuromuscular electrical stimulation.

**Table 4:** Summary of the literature examining muscle mass changes in patients who had femoral neck fractures and underwent postoperative rehabilitation SMI: Skeletal muscle mass index, ALM: Appendicular lean mass, FFM: Fat-free mass, ADL: Activities of daily living, BCAA: Branched-chain amino acid, BIA: Bioelectrical impedance analysis, DXA: Dual-energy X-ray absorptiometry, MRI: Magnetic resonance imaging.

Author (year)	Number of patients (n)	Rehabilitation	Outcome measurement	Method	Intervention period (months)	Values	Significant difference		
Ogawa et al. (2022) [9]	368	ADL and muscle strength training, 1–3 h/day	SMI	BIA	1.5	Median 5 kg/m^2 ^to 5.2 kg/m^2^	Increased significantly		
Chen et al. (2021) [8]	30	Not mentioned	Lower limb ALM	DXA	12	Mean 15.1 kg to 14.3 kg	Decreased significantly		
Briggs et al. (2018) [11]	17	Balance, resistance training, BCAA intake for 60 minutes 3 times/week for 12 weeks.	Quadriceps mass of fractured leg	MRI	3	Mean 36.3 cm^2 ^to 39.2 cm^2^	Increased significantly		
D'Adamo et al. (2014) [6]	155	Not mentioned	FFM	DXA	2	Mean 38 kg to 36.7 kg	No statistical analysis		
Binder et al. (2004) [5]	Control: 44	Resistance, balance training for at least three times/week for 12 weeks without a supervisor	FFM	DXA	6	Mean 39.7 kg to 40.5 kg	No statistical analysis		
	Intervention: 46	Same protocol with a supervisor				Median 41.7 kg to 42.9 kg			
Fox et al. (2000) [12]	205	Not mentioned	FFM	DXA	12	Median 39.3 kg to 37 kg	No statistical analysis		
Visser et al. (2000) [13]	90	Physical therapy for 12 months	ALM of non-fractured leg	DXA	12	Median 4.9 kg to 5 kg	No significant difference		
Nicholson et al. (1997) [10]	Control: 10	Not mentioned	FFM	BIA	1.5	Median 42.1 kg to 41.9 kg	No significant difference		
	Intervention: 20	A supervised seated exercise for 50 minutes, 24 sessions				Median 44.9 kg to 45.7 kg	No significant difference		

In the current study, anthropometric measurements at one and six weeks postoperatively were compared. The results showed an increase in lower limbs' skeletal muscle mass and a decrease in upper limbs' skeletal muscle mass and body weight. Total skeletal muscle mass and fat mass remained unchanged. In a study that reported an increase in skeletal muscle mass using bioelectrical impedance analysis as a measurement method, skeletal muscle index was compared between admission and discharge in 368 patients with hip fractures aged 80 years or older who were transferred to a rehabilitation hospital after surgery, where they underwent 1-3 hours of rehabilitation per day [9]. The authors reported an increase in skeletal muscle index from 5.0 kg/m^2^ at admission and 5.2 kg/m^2^ at discharge [9]. They concluded that it takes about 15 hours of exercise a week for 70 days to achieve these results [9]. In the current study, we observed an increase in lower limb skeletal muscle mass with a much shorter program of 14-21 hours of exercise per week for 42 days in a sample of patients in a similar age group. This result was considered to indicate the success of our program, as described above. In addition, total skeletal muscle mass did not change in this study, which we considered to be caused by a decrease in upper limbs and trunk skeletal muscle mass. It is possible that the amount of training for the upper limbs and trunk was less than the amount of training for the lower limbs in our hospital. Therefore, we aim to further increase the amount of skeletal muscle mass throughout the body by increasing the amount of training in the future.

In the current study, muscle strength was then evaluated in terms of grip strength of the dominant hand and knee extension muscle strength on the healthy and affected sides. Grip strength was maintained and knee extensor strength was increased on the healthy and affected sides.

According to a previous report [24], starting 3-4 weeks after surgery, lower limbs resistance training for three days per week for 12 weeks increased knee extensor strength from 65 N ± 17 to 78 N ± 13 (p = 0.011) on the healthy side and 41 N ± 15 to 66 N ± 11 (p = 0.006) on the affected side. Our results suggest that although the training was shorter (six weeks), the concentrated training for 5-7 days per week was able to increase muscle strength on both sides with equal or greater effectiveness.

Finally, the ability to perform ADL and walking ability were assessed in this study. All patients exhibited improved motor FIM scores at discharge, 66 days (IQR, 51-91) after surgery, compared with those at the time of injury or one week postoperatively. However, only 52.9% of patients exhibited recovery of walking ability that was equal to or better than that before the injury. In a study in which patients with hip fractures aged 70 years or older were followed up for three months after surgery, 59.1% of patients reported that their ADL was lower than before the injury [25]. Furthermore, in a study that investigated mobility disability among 184 patients who were independent regarding ADLs before hip fracture injury, 51.5% had a disability at three months and 42.9% at six months [2]. Although these studies have shown that the ability to perform ADL and mobility improve over time, the problem remains that approximately half of the patients after hip fractures remain impaired in ADL. Despite the short duration of our study compared with these studies, the rate of improvement in walking ability was equal or greater. Therefore, we considered that our results may have been better than those reported in previous studies in terms of long-term ADL improvement rates.

Our study had several limitations that should be considered. First, the number of patients was small. In the current study, we recruited 35 cases, but only approximately half of these (17 cases) were followed up for six weeks. Of the 18 patients, two were discharged, 14 were transferred to another rehabilitation hospital, and two dropped out due to worsening general conditions. Future research will need to increase the number of cases. In addition, it is important to consider how each surgical method, cognitive function, and nutritional status contributed. Second, the BIA measurements we used may have been inaccurate. Although it has been reported that BIA results are influenced by body water content, body water content was not measured in the current study. Increased muscle glycogen levels after exercise also led to increased body water content, which may have influenced the results of bioelectrical impedance analysis [26]. In the future, it will be necessary to simultaneously measure body water content. Third, the research period is short. In this study, body composition, grip strength, and lower limb muscle strength were measured six weeks after surgery. At our hospital, we set this period based on the fact that many patients are discharged from the hospital approximately 42 days after surgery. However, some past reports have shown that muscle hypertrophy occurs after six to seven weeks [27,28]. Therefore, future research should extend the intervention period further. Patients’ nutritional status was not assessed. We did not assess whether caloric intake was sufficient for the amount of exercise. In the future, it will be necessary to calculate the amount of energy required for each patient and measure the amount of food consumed. However, a major strength of this study is that, to our knowledge, it is the first report to demonstrate a postoperative rehabilitation program that increased lower limbs skeletal muscle mass without decrease in total skeletal muscle mass. At the same time, the observed improvements in muscle strength, the ability to perform ADL, and walking ability in the current study showed that our postoperative rehabilitation program was effective for some aspects of recovery in a relatively short period of time.

## Conclusions

We reported a detailed rehabilitation program for hip fracture patients, featuring early ambulation training and muscle strengthening exercises combined with neuromuscular electrical stimulation. Our rehabilitation program for hip fracture patients led to increased skeletal muscle mass of the lower limbs at six weeks postoperatively.

## References

[1] Hagino H (2012). Fragility fracture prevention: review from a Japanese perspective. Yonago Acta Med.

[2] Ouellet JA, Ouellet GM, Romegialli AM (2019). Functional outcomes after hip fracture in independent community-dwelling patients. J Am Geriatr Soc.

[3] Fukui N, Watanabe Y, Nakano T, Sawaguchi T, Matsushita T (2012). Predictors for ambulatory ability and the change in ADL after hip fracture in patients with different levels of mobility before injury: a 1-year prospective cohort study. J Orthop Trauma.

[4] Gimigliano F, Liguori S, Moretti A (2020). Systematic review of clinical practice guidelines for adults with fractures: identification of best evidence for rehabilitation to develop the WHO's Package of Interventions for Rehabilitation. J Orthop Traumatol.

[5] Binder EF, Brown M, Sinacore DR, Steger-May K, Yarasheski KE, Schechtman KB (2004). Effects of extended outpatient rehabilitation after hip fracture: a randomized controlled trial. JAMA.

[6] D'Adamo CR, Hawkes WG, Miller RR (2014). Short-term changes in body composition after surgical repair of hip fracture. Age Ageing.

[7] Iida H, Seki T, Sakai Y, Watanabe T, Wakao N, Matsui H, Imagama S (2021). Low muscle mass affect hip fracture treatment outcomes in older individuals: a single-institution case-control study. BMC Musculoskelet Disord.

[8] Chen YP, Kuo YJ, Hung SW (2021). Loss of skeletal muscle mass can be predicted by sarcopenia and reflects poor functional recovery at one year after surgery for geriatric hip fractures. Injury.

[9] Ogawa T, Sato K, Nakayama Y (2022). Factors associated with actual skeletal muscle mass increase during hip fracture rehabilitation of persons aged 80 and older. Arch Gerontol Geriatr.

[10] Nicholson CM, Czernwicz S, Mandilas G, Rudolph I, Greyling MJ (1997). The role of chair exercises for older adults following hip fracture. S Afr Med J.

[11] Briggs RA, Houck JR, LaStayo PC, Fritz JM, Drummond MJ, Marcus RL (2018). High-intensity multimodal resistance training improves muscle function, symmetry during a sit-to-stand task, and physical function following hip fracture. J Nutr Health Aging.

[12] Fox KM, Magaziner J, Hawkes WG (2000). Loss of bone density and lean body mass after hip fracture. Osteoporos Int.

[13] Visser M, Harris TB, Fox KM (2000). Change in muscle mass and muscle strength after a hip fracture: relationship to mobility recovery. J Gerontol A Biol Sci Med Sci.

[14] Brunner LC, Eshilian-Oates L, Kuo TY (2003). Hip fractures in adults. Am Fam Physician.

[15] Charlson ME, Pompei P, Ales KL, MacKenzie CR (1987). A new method of classifying prognostic comorbidity in longitudinal studies: development and validation. J Chronic Dis.

[16] Braid V, Barber M, Mitchell SL, Martin BJ, Granat M, Stott DJ (2008). Randomised controlled trial of electrical stimulation of the quadriceps after proximal femoral fracture. Aging Clin Exp Res.

[17] Conley CE, Mattacola CG, Jochimsen KN, Dressler EV, Lattermann C, Howard JS (2021). A comparison of neuromuscular electrical stimulation parameters for postoperative quadriceps strength in patients after knee surgery: a systematic review. Sports Health.

[18] Lahav Y, Goldstein N, Gepner Y (2021). Comparison of body composition assessment across body mass index categories by two multifrequency bioelectrical impedance analysis devices and dual-energy X-ray absorptiometry in clinical settings. Eur J Clin Nutr.

[19] Martin JA, Ramsay J, Hughes C, Peters DM, Edwards MG (2015). Age and grip strength predict hand dexterity in adults. PLoS One.

[20] Bai D, Tokuda M, Ikemoto T (2021). Effect of types of proximal femoral fractures on physical function such as lower limb function and activities of daily living. Phys Ther Res.

[21] Yoon SH, Kim BR, Lee SY, Beom J, Choi JH, Lim JY (2021). Influence of comorbidities on functional outcomes in patients with surgically treated fragility hip fractures: a retrospective cohort study. BMC Geriatr.

[22] Holden MK, Gill KM, Magliozzi MR, Nathan J, Piehl-Baker L (1984). Clinical gait assessment in the neurologically impaired. Reliability and meaningfulness. Phys Ther.

[23] Granger CV, Hamilton BB, Linacre JM, Heinemann AW, Wright BD (1993). Performance profiles of the functional independence measure. Am J Phys Med Rehabil.

[24] Hauer K, Specht N, Schuler M, Bärtsch P, Oster P (2002). Intensive physical training in geriatric patients after severe falls and hip surgery. Age Ageing.

[25] van der Sijp MP, van Eijk M, Niggebrugge AH, Putter H, Blauw GJ, Achterberg WP (2021). Prognostic factors for short-term recovery of independence in a multistate model for patients with a hip fracture. J Am Med Dir Assoc.

[26] Consitt LA, Dudley C, Saxena G (2019). Impact of endurance and resistance training on skeletal muscle glucose metabolism in older adults. Nutrients.

[27] Damas F, Libardi CA, Ugrinowitsch C (2018). The development of skeletal muscle hypertrophy through resistance training: the role of muscle damage and muscle protein synthesis. Eur J Appl Physiol.

[28] Schoenfeld BJ (2010). The mechanisms of muscle hypertrophy and their application to resistance training. J Strength Cond Res.

